# Microbial Communities in a High Arctic Polar Desert Landscape

**DOI:** 10.3389/fmicb.2016.00419

**Published:** 2016-03-31

**Authors:** Clare M. McCann, Matthew J. Wade, Neil D. Gray, Jennifer A. Roberts, Casey R. J. Hubert, David W. Graham

**Affiliations:** ^1^School of Civil Engineering and Geosciences, Newcastle UniversityNewcastle upon Tyne, UK; ^2^Department of Geology, University of Kansas, LawrenceKS, USA; ^3^Energy Bioengineering and Geomicrobiology, University of Calgary, CalgaryAB, Canada

**Keywords:** polar soils, biogeochemistry, microbial diversity, ecology, phosphorus

## Abstract

The High Arctic is dominated by polar desert habitats whose microbial communities are poorly understood. In this study, we used next generation sequencing to describe the α- and β-diversity of microbial communities in polar desert soils from the Kongsfjorden region of Svalbard. Ten phyla dominated the soils and accounted for 95% of all sequences, with the *Proteobacteria, Actinobacteria*, and *Chloroflexi* being the major lineages. In contrast to previous investigations of Arctic soils, relative *Acidobacterial* abundances were found to be very low as were the Archaea throughout the Kongsfjorden polar desert landscape. Lower *Acidobacterial* abundances were attributed to characteristic circumneutral soil pHs in this region, which has resulted from the weathering of underlying carbonate bedrock. In addition, we compared previously measured geochemical conditions as possible controls on soil microbial communities. Phosphorus, pH, nitrogen, and calcium levels all significantly correlated with β-diversity, indicating landscape-scale lithological control of available nutrients, which in turn, significantly influenced soil community composition. In addition, soil phosphorus and pH significantly correlated with α-diversity, particularly with the Shannon diversity and Chao 1 richness indices.

## Introduction

Polar soil microbiology has been understudied in comparison to temperate biomes (Bell et al., 2013; Blaud et al., 2015). However, research on these soils is intensifying in response to concerns over climate warming (Singh et al., 2010). Increasing regional temperatures will result in the thawing of permafrost, releasing previously sequestered carbon for microbial degradation (Davidson and Janssens, 2006). Critically, the Arctic region stores almost half of the world’s terrestrial carbon (Tarnocai et al., 2009), and microbial mineralization of this carbon has the potential to greatly increase greenhouse gas emissions (Schuur et al., 2009). Therefore, developing a deeper understanding of baseline Arctic soil microbial communities and their environmental controls is essential for anticipating and monitoring change in this dynamic region.

Studies of Arctic soil microbial communities to date have largely focused on comparisons of communities in active layers and underlying permafrost (Yergeau et al., 2010); soil chronosequences (Schutte et al., 2009); and most intensively, biogeochemical processes occurring in tundra soils (Høj et al., 2005; Graef et al., 2011; Tveit et al., 2013). Tundra soils have been a past focus of research as they are assumed to release the largest amount of greenhouse gases in a warming climate (Tarnocai et al., 2009). In contrast, polar desert soil communities have received less attention, despite accounting for 1,358,000 km^2^ of the Arctic region (Walker et al., 2002) and the fact that greenhouse gas emissions from such soils are comparable to tundra landscapes (Brummell et al., 2014). The soil microbial communities in Antarctic polar deserts are better described (Niederberger et al., 2008; Yergeau et al., 2008; Geyer et al., 2014), but currently little information exists on these environments in the Arctic.

Recently, a pan-Arctic biogeographical study of microbial communities associated with Arctic tundra soils showed that their dominant phyla were similar to those found at lower latitudes and were structured according to variation in soil pH (Chu et al., 2010). This survey included geographically diverse sites that were often more than 100 km apart and spanned the Canadian, Siberian, and European High Arctic. However, the majority of the studied soils had pH values below 6.0. More recently, we took a more local landscape approach, studying of the influence of geochemical factors on bacterial abundances across 13 soils from the Kongsfjorden area of the Norwegian High Arctic, which included seven polar desert soils (Gray et al., 2014). In this landscape, weathering of carbonates and mica schists has resulted in soils that predominately have pH values above 6.0 (Mann et al., 1986; Gray et al., 2014). Further, it was found that differences in soil mineralogy defined local variations in pH that, in turn, influenced soil phosphorous (P) conditions (Gray et al., 2014). Across this landscape, available soil P was limiting and variation in P significantly correlated with bacterial and Type I methanotroph 16S rRNA gene abundances.

In this current study, we have applied high-throughput amplicon sequencing to these soils with the aim of characterizing the microbial diversity (α-diversity) and community composition (β-diversity) in the seven previous polar desert soils plus two supplementary Kongsfjorden soils for comparative assessment with previous global and pan-Arctic data. An obvious question arising from our previous work (Gray et al., 2014) was whether measured geochemical variables that correlated with microbial abundances also influenced overall soil microbial diversity in the polar desert soils.

## Materials and Methods

### Study Sites, Sampling, and Edaphic Characterization

Soil cores were collected from the southern and northern shores of Kongsfjorden, Svalbard in late August 2010, when the active layer depths were at their maximum (Gray et al., 2014). Cores were taken from the top 10 cm of the soil profile in areas generally void of vegetation. This approach ensured the same depth range was obtained for all samples which bracketed the ground surface and the anaerobic–aerobic interface. At each site, three soil cores were recovered using sterile plastic syringes with the luer-end removed. On return to the NERC field laboratory, cores were frozen immediately and subsequently shipped frozen to the UK where they were stored at –20°C until further analysis.

Seven of the soils studied were from sites that represent polar deserts in the Kongsfjorden region (SE, GS2, OS, BR1, NL2, SL2, and BZ2). A map showing these sampling locations is available in Gray et al. (2014). These sites were chosen to represent polar desert and semi-desert vegetation types, previously described in detail by Wookey et al. (1995), Walker et al. (2005), and Madan et al. (2007), within the *Dryas octopetala* zone of Svalbard, defined as bioclimatic subzone B and vegetation type P1. The seven soils were also chosen to span the major lithological and geochemical gradients across the region, which have resulted from differential weathering of metamorphic carbonate and aluminosilicate bedrock (Supplementary Figure 1). Specifically, our previous work had found this lithology significantly influenced local soil pH and P, apparently due to differing inputs of dolomite [MgCa(CO_3_)_2_] and calcite (CaCO_3_), during pedogenesis.

Two additional sites were included to provide a contrast to the polar desert soils in the region. This included one site from a glacial moraine (BBM) and another from an organic-rich tundra (SN), which has been studied in detail (Tveit et al., 2013). The glacial moraine was not considered a polar desert soil as it comprised rock and soil debris from the foot of a glacier that has been transported by the glacier and its meltwater rather than weathered *in situ* from the bedrock. Geochemical characteristics of the first eight soils are summarized in Gray et al. (2014), whereas new data for the tundra soil (SN) are provided in the Supplementary Material. Soil nutrients, metals, mineralogy, and isotopic analytical methods are reported in Gray et al. (2014). Synoptic data describing the geochemical characteristics of the seven polar desert soils studied herein are provided in **Table 1**.

**Table 1:** Average measured geochemical characteristics of the seven polar desert soils in Kongsfjorden, Svalbard.

Geochemical variable	Average ±*SE*
Moisture content (%)	66.4 ± 30.6
pH	6.88 ± 0.49
SOM (%)	9.00 ± 2.37
TOC (%)	5.22 ± 1.38
Nitrite (mM NO_2_^-^ g^-1^ ^drysoil^)	BD
Nitrate (mM NO_3_^-^ g^-1drysoil^)	0.80 ± 0.23
Ammonium (mM NH_4_^+^ g^-1drysoil^)	1.27 ± 0.43
Total Kjeldahl Nitrogen (mg/kg)	2280 ± 810
Total Phosphorus (mg/kg)	450 ± 51.3

### Barcoded Pyrosequencing

Genomic DNA was extracted from 0.5 g of soil using the FastDNA Spin Kit for soil (MP Biomedicals, UK), and a FastPrep Ribolyser (MP Biomedicals, France), according to the manufacturer’s protocol. PCR amplifications were carried out with universal primer pair F515 and R926 (positions 515 to 926 in the V4–V5 region; *Escherichia coli* numbering), which target both Bacteria and Archaea. Primer F515 was adapted to include a 25 bp ‘A’ Adaptor followed by a 12 bp unique barcode and a 2 bp (CA) linker attached to the 5′ end. Primer R926 was modified to contain a 25 bp ‘B’ Adaptor attached to the 5′ end.

PCR reactions were carried out with a total volume of 25 μl, containing 10 μM of each primer, 10 mM of deoxynucleoside triphosphates (dNTPs), 2.5 μl of FastStart HiFi 10x Buffer #2 (Roche Diagnostics Ltd., UK), 0.25 μl of FastStart HiFi Polymerase (5 U/μl; Roche Diagnostics Ltd., UK), 18.75 μl of molecular biology grade water (Sigma–Aldrich, UK) and 1 μl of neat DNA. PCRs were then subjected to an initial denaturation at 95°C for 4 min; 28 cycles of denaturation at 95°C for 1 min; annealing at 58°C for 45 s; and extension at 72°C for 1 min; followed by a final extension at 72°C for 8 min in an automated thermal cycler Techne TC-5000 (Bibby Scientific, UK).

Ten PCR amplicons per sample were combined to allow for sufficient recovery after a gel extraction clean up. For purification, PCR products were first purified using a QIAquick PCR Purification Kit (Qiagen, UK) followed by a QIAquick Gel Extraction Kit (Qiagen, UK). The concentration of DNA in purified PCR samples was quantified by UV-Vis spectrophotometry using a Nanodrop 2000 (Thermo scientific, Wilmington, DE, USA) to ensure amounts sufficient for sequencing. Equimolar pooling of the differentially barcoded amplicons and subsequent sequencing was carried out by NewGene (International Centre for Life, Newcastle upon Tyne, UK), using the Roche 454 sequencing GS FLX Titanium Series.

### Processing of Pyrosequencing Data and Statistical Analysis

Sequences were processed using the QIIME v1.4.0 open-source software package (Caporaso et al., 2010), previously described in detail by McCann et al. (2015). Libraries were rarefied to 3946 sequences per sample at a truncated read length of 400 bp to ensure that differences in sequencing depth did not bias diversity estimations. The sequences have been deposited in the NCBI’s Sequence Read Archive (SRA) and are available under the BioProject ID PRJNA308796.

All α-diversity estimates were generated in QIIME. Overall bacterial α-diversity was estimated using a taxonomic [Shannon–Weaver index, *H′* (Shannon and Weaver, 1949)] and a phylogenetic [Faiths phylogenetic diversity, PD, (Faith, 1992)] method. The Shannon index is a phylotype-based approach constructed using OTU groupings (Ludwig, 1988). In contrast, Faith’s index denotes the proportion of branch lengths represented by one community relative to the total of all branch lengths across all communities, providing a relative estimate of the overall phylogenetic (tree) breadth within a given community (Lozupone and Knight, 2008; Fierer et al., 2009).

In addition to Shannon and Faith, we also determined the non-parametric abundance-based Chao 1 (*S*_chao_) indicator to estimate species richness (Chao, 1984). This approach is based on the concept that rare species carry the most information about the number of missing species, therefore *S*_chao_ only uses the numbers of singletons and doubletons to obtain a total OTU estimate (Gotelli and Chao, 2013). Evenness was calculated using the Equitability metric (*E*_D_), which assumes a value between 0 and 1, with 1 being complete evenness (Shannon and Weaver, 1949). In addition, we estimated the sampling effort required to obtain 90% of the true taxonomic diversity following the method of Quince et al. (2008). Environmental data were imported into R and the ordistep function in the *vegan* package (Oksanen et al., 2012) was used to perform automatic stepwise model generation with redundancy analysis (RDA).

Univariate analysis was carried out using SPSS 21 for Windows (SPSS, Inc. Chicago, IL, USA). Correlations between α and β-diversity estimates and all other variables were carried out using curve estimation regression of linear and quadratic models with the model chosen based on highest *r*-values. Differences of *p* ≤ 0.05 were defined as statistically significant.

## Results

### β-Diversity

The dominant bacterial phyla in the seven polar desert soils, which captured 96% of all sequences, included the *Proteobacteria* (32.0 ± 12.1), *Actinobacteria* (21.6 ± 8.16), *Chloroflexi* (12.8 ± 4.85), *Acidobacteria* (5.87 ± 0.56), *Bacteroidetes* (5.44 ± 2.06), *Planctomycetes* (5.22 ± 1.97), *Cyanobacteria* (4.50 ± 1.70), *Gemmatimonadetes* (3.90 ± 1.47), *Verrucimicrobia* (2.07 ± 0.78), unclassified bacteria (1.49 ± 0.56) and *Firmicutes* (1.44 ± 0.54). Thirty-nine bacterial phyla were identified in total (Supplementary Table 2). At the finest level of OTU resolution, an unclassified genus from the family *Gaiellaceae* was the most abundant sequence type in the polar desert soils (4.72 ± 1.71%, **Table 2**). Archaeal populations represented only 0.37 ± 0.13% of all sequences in the polar desert soils of which the *Thaumarchaeota* phylum (genus *Nitrososphaera*) dominated representing 77.5% of the total community (**Table 3**). The *Crenarchaeota* comprised sequences of the miscellaneous crenarchaeotal group (MCG; 6.48%), whereas, the *Euryarchaeota* comprised three distinct classes, namely, the *Methanobacteria* (8.53%), *Methanomicrobia* (3.22%) and the *Parvarchaea* (4.29%).

**Table 2:** List of the most abundant bacterial groups (%) classified to the genus level, identified according to the results if the 16S rRNA pyrosequencing, in the polar desert soils of Kongsfjorden, Svalbard.

Phylum	Class	Order	Family	Genus	
Other	Other	Other	Other	Other	1.49 ± 0.26
*Actinobacteria*	*Actinobacteria*	*Actinomycetales*	*Intrasporangiaceae*	–	3.11 ± 0.80
*Actinobacteria*	*Thermoleophilia*	*Gaiellales*	*Gaiellaceae*	–	4.72 ± 1.71
*Actinobacteria*	*Thermoleophilia*	*Solirubrobacterales*	*–*	–	2.86 ± 0.91
*Bacteroidetes*	*Sphingobacteriia*	*Sphingobacteriales*	*Chitinophagaceae*	–	1.53 ± 0.25
*Chloroflexi*	*Anaerolineae*	*SBR1031*	*A4b*	–	2.58 ± 1.03
*Chloroflexi*	*Ellin6529*	*–*	*–*	–	3.86 ± 0.89
*Proteobacteria*	*Betaproteobacteria*	Other	Other	Other	3.05 ± 1.64
*Proteobacteria*	*Betaproteobacteria*	*Burkholderiales*	*Comamonadaceae*	Other	2.34 ± 0.61
*Proteobacteria*	*Gammaproteobacteria*	*Xanthomonadales*	*Xanthomonadaceae*	Other	1.57 ± 0.68

**Table 3:** List of the Archaeal taxonomic groups, identified according to the results if the 16S rRNA pyrosequencing, composing the archaeal communities in the polar desert soils of Kongsfjorden, Svalbard.

Phylum	Class	Order	Family	Genus	
*Crenarchaeota*	MCG		*–*	*–*	6.48 ± 0.01
*Thaumarchaeota*		*Nitrososphaerales*	*Nitrososphaeraceae*	*Nitrososphaera*	77.5 ± 0.16
*Euryarchaeota*	*Methanobacteria*	*Methanobacteriales*	*Methanobacteriaceae*	*Methanobacterium*	8.53 ± 0.03
*Euryarchaeota*	*Methanomicrobia*	*Methanosarcinales*	*Methanosarcinaceae*	*Methanosarcina*	1.32 ± 0.00
*Euryarchaeota*	*Methanomicrobia*	*Methanocellales*	*–*	*–*	1.90 ± 0.01
*Parvarchaeota*			*–*	*–*	4.29 ± 0.01

*Results are expressed in % with respect to the total archaeal community.*

The seven polar desert soils had very similar community compositions to the tundra and moraine samples at the phylum level (see Supplementary Figure 3). The only substantial difference observed was in the proportion of *Cyanobacterial* sequences, which were in much larger abundance in the desert soils than the moraine or tundra comparators (see Supplementary Table 3).

### Geochemical Variables and β-Diversity

Both univariate and multivariate statistical analyses were performed to assess potential relationships between geochemical variables and the microbial β-diversity metrics. Univariate analysis of the relative abundance of the dominant phyla in the polar desert soils showed significant linear relationships between the *Proteobacteria* and soil pH (*r* = 0.81, *p* = 0.03), and the *Proteobacteria* and soil P (*r* = 0.86, *p* = 0.01). Further, the relative abundance of unclassified bacteria in the polar desert soils displayed positive trends with moisture content (*r* = 0.96, *p* < 0.01), TOC (*r* = 0.73, *p* < 0.1) and total kjeldahl nitrogen (TKN; *r* = 0.71, *p* < 0.1). No other variables showed significant correlations (*p* > 0.05) or major trends with the relative abundance of the other dominant or lesser phyla.

Multivariate RDA showed that no combination of measured variables could explain the variation for scaled (relative abundance) data. However, for unscaled data (i.e., presence/absence), P, Ca, and TKN combined highly significantly explained differences among the polar desert soils (*p* < 0.01). No other measured variables provided significant relationships (*p* < 0.05).

### α-Diversity

Measures of bacterial diversity, richness and evenness were similar across the seven polar desert soils when normalized to the same number of sequence reads; i.e., 3,946 sequences per soil (**Table 4**). The soil series displayed the following mean diversity values: *H′* = 8.28 ± 0.29, PD = 74.3 ± 8.14, *S*_chao_ = 1890 ± 118, *S* = 870 ± 119, and *E*_D_ = 0.83 ± 0.02. Sites BZ2 and SE had the highest and lowest diversity and evenness metrics among the samples, respectively, with site SE consistently showing the lowest α-diversity metrics relative to other sites (Supplementary Table 4).

**Table 4:** Average measured diversity metrics of the seven polar desert soils in Kongsfjorden, Svalbard.

	Average ±*SE*
Shannon (*H*′)	8.28 ± 0.29
Faith (PD)	80.8 ± 6.94
Chao 1 (*S*_chao_)	1890 ± 216
Observed species (*S*)	948 ± 96.6
Equitability (*E*_D_)	0.84 ± 0.01

The sequencing depth of our amplicon libraries is comparable to other studies that used pyrosequencing to estimate soil biodiversity, such as Lauber et al. (2009, 2013) and Chu et al. (2010). However, we presume that the ‘true’ diversity is not estimated well by the non-parametric α-diversity metrics employed. To better define the level of sequencing needed to capture the actual bacterial diversity and evenness, we used a parametric Bayesian approach [based upon taxa abundance distribution modeling as per Quince et al. (2008)]. Using this method, we estimated that between 10 and 49 additional runs of a Roche FLX genome sequencer would be needed to capture 90% of the true diversity in the seven polar desert soil samples (see Supplementary Tables 5–8). As such, the maximum diversity in these soils may be higher than we report (Supplementary Table 2).

The tundra soil (SN) showed similar diversity metrics to the polar desert soils (*H′* of 8.7) and was the second most diverse site according to all metrics, except non-parametric richness [*S*_chao_] (Supplementary Table 4). In contrast, the glacial moraine soil showed very low diversity indices with *S*_chao_, *S*, and Faith’s PD being almost half that of the polar desert and tundra soils. Estimates of total diversity for the moraine soil suggested it was under sequenced and would require an additional 464 runs to capture 90% of the total diversity (Supplementary Table 5).

### Geochemical Variables and α-Diversity

**FIGURE 1:**
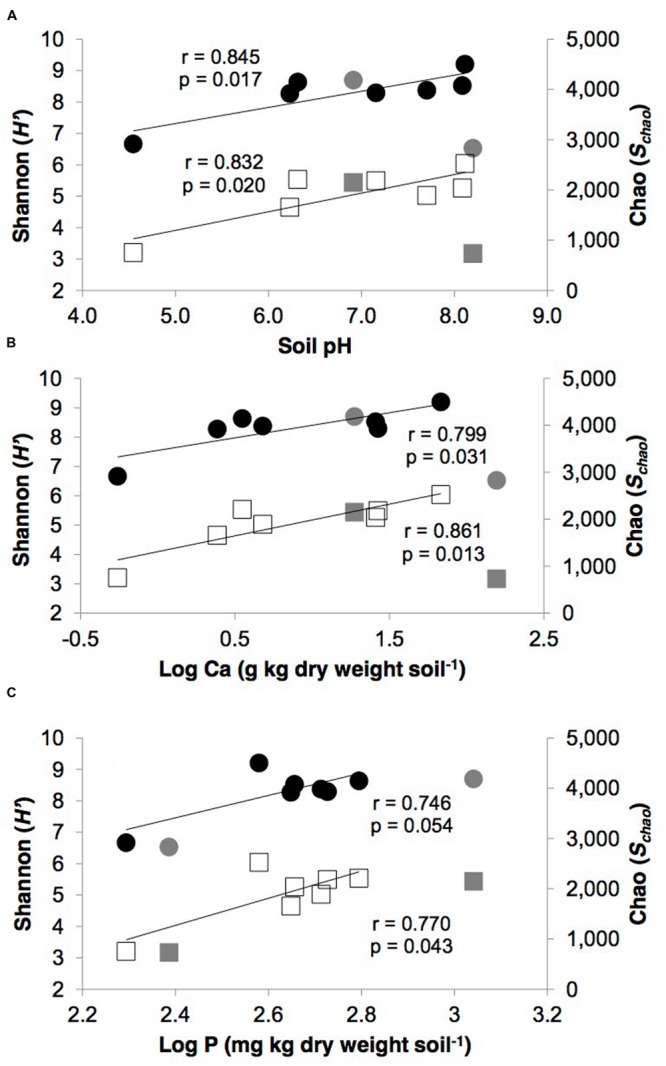
**Relationships between measures of taxonomic diversity (Shannon, *H′*, closed circles) and richness (Chao, *S*_chao_, open squares) versus polar desert soil **(A)** pH, **(B)** calcium, **(C)** phosphorus.** Gray circles and squares represent the tundra (SN) and glacial moraine (BBM) soil samples, which were not included in the statistical analysis. Diversity metrics were based upon 10 random iterations of the pyrosequencing dataset data rarefied to 3,946 reads per library. The *p*-values indicate the significance of linear (*r*) correlation coefficients for pairs of variables (SPSS Statistics version 21; IBM).

The pH and calcium levels in the polar desert soils showed significant linear correlations with both taxonomic diversity (*H′*) and richness (*S*_chao_; *p* ≤ 0.05, **Figure 1**). Bayesian parametric total diversity estimates also displayed significant linear relationships with calcium (*r* = 0.805, *p* = 0.029). In addition, soil P displayed significant linear relationships with *H′* (*r* = 0.744, *p* = 0.021) and *S*_chao_ (*r* = 0.744, *p* = 0.022). No other edaphic parameters significantly correlated with the diversity metrics for these soils (*p* > 0.05).

## Discussion

### Dominant Bacterial Phyla in the Kongsfjorden Polar Desert Soils

**FIGURE 2:**
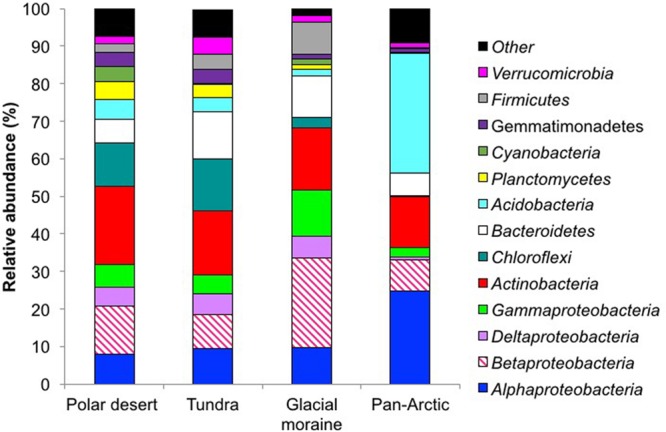
**Relative abundances of the dominant bacterial phyla in the polar desert soils (*n* = 7), a glacial moraine (*n* = 1) and the tundra (*n* = 1) soil.** These have been compared to the dominant phyla from 29 soils across the Arctic by Chu et al. (2010). All abundances are based upon the proportional frequency of sequences that could be identified at the phylum level.

The dominant bacterial phyla found in the Kongsfjorden polar desert soils are consistent with the most abundant bacterial phyla observed in a global meta-analysis of soils (Janssen, 2006). This conclusion supports the idea that gross bacterial communities in the Arctic are not too different from temperate biomes at the phylum level (Chu et al., 2010). Nevertheless, this study, which was conducted over a 100 km^2^ landscape, displayed notable differences. Specifically, the *Planctomycetes, Cyanobacteria, Gemmatimonadetes, Firmicutes, Chloroflexi*, and *Verrucimicrobia* were all at higher relative abundances than the levels reported by Chu et al. (2010), (see **Figure 2**) being closer to the global averages summarized by Janssen (2006).

Interestingly, the *Proteobacteria* were the most dominant phyla in the Kongsfjorden polar desert soils, which differs from studies of Canadian, Alaskan, and Siberian Arctic soils that report *Acidobacteria* as a dominant phylum (Neufeld and Mohn, 2005; Wallenstein et al., 2009; Campbell et al., 2010; Chu et al., 2010; Rawat et al., 2012; Männistö et al., 2013). However, the dominance of the *Proteobacteria* over the *Acidobacteria* across the region is consistent with previous sequencing studies of Kongsfjorden tundra soil (Lee et al., 2013; Tveit et al., 2013). In addition, this dominance is consistent with measured differences in the soil pH across the sites. Such a pH-related selection is not surprising when one considers *Acidobacteria* usually have pH growth optima in the range of 3–6 (Jones et al., 2009). The pH values of the polar desert soils of the Kongsfjorden landscape were circumneutral with only one value below 6 as dictated by the differential weathering inputs from carbonate rocks (e.g., dolomite and calcite), which likely selects against *Acidobacterial* strains. In comparison, the pan-Arctic survey studied soils with pH values mostly below 6 (Chu et al., 2010).

A caveat regarding the utility of comparing the dominant phyla identified in this study with those identified by Chu et al. (2010) is that different primer pairs were used to generate the sequence libraries. Specifically, Chu et al. (2010) used the primer pair 27F and 338R (positions 27 to 338 in the V1–V3 region; *Escherichia coli* numbering), whereas the current study employed the primer pair F515 and R926 (positions 515 to 926 in the V4–V5 region; *Escherichia coli* numbering). An analysis of the coverage of these both these primer pairs performed using the RDP probe match analysis tool indicated that the primer pair 27F and 338R used by Chu et al. (2010) targeted only 11% of total bacterial sequences, 14% of *Proteobacterial* sequences, 17% of *Acidobacterial* sequences and 13% of *Cyanobacterial* sequences of the full length sequences listed in the RDP database. In contrast the primer pair F515 and R926 used here targeted 88% of total bacterial sequences, 93% of *Proteobacterial* sequences, 94% of *Acidobacterial* sequences, and 94% of *Cyanobacterial* sequences.

Another feature of our investigation was that the *Cyanobacteria* comprised 4.50% of the total bacterial community in the polar desert soils, a value considerably larger than the 0.24% reported for the more intensively studied Pan-Arctic tundra soils of Chu et al. (2010). This dominance of *Cyanobacteria* may be a specific feature of our desert soils as they only represented 0.24% in the one tundra soil included in our survey. Regardless of the potential variation in sequence classification caused by different amplification protocols, such an issue does not explain differences in *Cyanobacteria* observed between the polar desert soils and the of Kongsfjorden tundra soil, which was accomplished using the same primer sets. Furthermore, *Cyanobacteria* were not reported in other sequencing studies of Kongsfjorden tundra (Lee et al., 2013; Tveit et al., 2013).

The *Cyanobacteria* have been found in many Arctic soils and have been reported to fix atmospheric N_2_ and CO_2_, especially in nutrient-poor soils (Vincent, 2002; Stewart et al., 2011; Michaud et al., 2012). For instance, *Leptolyngbya* spp., which represented on average 33.0% of the total *Cyanobacteria* in our polar desert soils (see Supplementary Table 7), have been shown to fix N_2_ and appear to be widespread in Svalbard based on their identification in other sequencing studies in the wider region (Kaštovská et al., 2005; Turicchia et al., 2005; Stibal et al., 2009; Schostag et al., 2015). On this basis, *Cyanobacteria* may play an important role in nutrient cycling in the Kongsfjorden polar desert soils, which have been previously reported as N- and/or P-limited (Gray et al., 2014). In comparison, the *Cyanobacteria* identified in the single Kongsfjorden tundra soil were dominated by the *Chloroplast* class, comprising 32.2% of the total *Cyanobacterial* community in this soil (Supplementary Table 7).

At the finest level of OTU resolution, an unclassified genus from the family *Gaiellaceae* was the most abundant taxon in the polar desert soils (4.72 ± 1.71, **Table 2**). This novel family has only been identified by culture-independent techniques and remains poorly understood, although members have been shown to be strict aerobes and chemoorganotrophs (Albuquerque and Costa, 2014). To our knowledge, this is the first report of *Gaiellaceae* in Svalbard.

### Dominant Archaeal Phyla in the Kongsfjorden Polar Desert Soils

In comparison to the bacterial population, the archaeal community was present at a low relative abundance (0.37%), in relation to the reported global soil average of 2.0% (Bates et al., 2011). This difference either suggests Archaea are less abundant in this polar desert environment or that our near-surface sampling did not capture the whole archaeal community. A similar low abundance of Archaea in Kongsfjorden has been reported previously for tundra soils [0.01–0.13%; (Tveit et al., 2013)] that were dominated by methanogens. In contrast, the archaeal communities in the polar desert soils were dominated by *Nitrososphaera*, which is a genus of ammonia oxidizing archaea (AOA). This dominance has also been found by Hayashi et al. (2015) in polar desert soils sampled from a single location in Kongsfjorden. In their study, six major OTUs were identified from *amoA* gene pyrosequencing, five of which were grouped within the *Nitrososphaera* cluster of archaeal ammonia oxidizers considered a group with zero or very low ammonia oxidation rates. It is now clear from this current study that this dominance of *Nitrososphaera* extends across the whole Kongsfjorden landscape. However, Hayashi et al. (2015) concluded from their single soil location that ammonia oxidation was driven mainly by psychrotolerant ammonia oxidizing bacteria also identified in their study.

In addition, to the presence of AOA in our study, the MCG was detected in the polar desert soils. The MCG have been hypothesized to have a significant role in dissimilatory methane oxidation (Biddle et al., 2006). For the minor component of the Archaea that were affiliated to the *Euryarchaeota* in the polar desert soils, sequences were either related to methanogenic *Methanobacteriaceae* or *Methanomicrobia.* These are hydrogenotrophic methanogens (producing CH_4_ from H_2_ and CO_2_) that, as described above, have been identified in Kongsfjorden tundra soils (Tveit et al., 2013). A further interesting finding in the polar desert soils was the presence of *Parvarchaea*, which accounted for 4.29% of the total archaeal community. The *Parvarchaea* are among the smallest cellular life forms known (<500 nm diameter) and are still poorly described. These rare Archaea have been found in permafrost soils of the Alaskan Arctic (Kao-Kniffin et al., 2015), but to our knowledge this is the first report of their presence in the Norwegian Arctic.

### The Effect of Variation in Geochemistry on the Diversity in the Kongsfjorden Polar Desert Soils

The presence and absence of OTUs between different soils (commonly termed as β-diversity) was primarily driven by a combination of the variation in Ca, pH, P, and TKN. Yet relationships between specific OTUs and measured geochemical variables were less clear, with only soil pH and P showing significant linear correlations with the relative abundances of the *Proteobacteria* the most dominant phylum. Both N and P have been suggested as a limiting nutrients in the Kongsfjorden region by Gray et al. (2014). In our previous work, we identified a strong intrinsic relationship between Ca and P levels in the Kongsfjorden soils. Specifically, P levels varied with Ca (a proxy for soil carbonates and pH) in an inverted U-shaped pattern where P levels were highest at intermediate Ca concentrations (Gray et al., 2014). Further, bacterial gene abundances showed the same relationship with maximum abundances prevailing at intermediate Ca values. In turn, bacterial abundances significantly correlated with soil P, which suggested potential P-limitation. The results in the current study add to this knowledge and suggest that P, Ca, and pH not only influence bacterial abundance, but also community composition.

Soil P is gaining increasing attention in polar settings and its role as an environmental control on such soils has been observed by others (Siciliano et al., 2014). For example, P was found to be potentially limiting in High Arctic soil chronosequences (Schutte et al., 2009); for plant growth (Wookey et al., 1995; Madan et al., 2007); and in glacier bacterial populations (Mindl et al., 2007; Stibal et al., 2009). Such effects are not likely limited to the Kongsfjorden region. However, because P-limitation appears widespread in polar latitudes (Zamin and Grogan, 2012). P-availability may be less significant in more temperate soils where moisture availability and inputs of plant-derived organic carbon are major drivers of bacterial diversity (Fierer and Jackson, 2006). Critically, its limited availability in polar latitudes may impact on nature’s response to a changing climate.

The effect of the variation in geochemical variables was more apparent for α-diversity than β-diversity in the desert soils. Both soil pH and Ca showed significant linear correlations with taxonomic diversity (*H′*) and richness (*S*_chao_). As our previous work showed soil pH and Ca levels in the Kongsfjorden region are driven by differential weathering of carbonate-rich bedrock, which in turn, impacts relative soil pH levels, therefore it is not unexpected that both variables showed significant correlations. These findings are consistent with previous studies that suggested soil pH was the best predictor of α-diversity in Pan-Arctic soils (Chu et al., 2010), and also at regional and continental scales (Fierer and Jackson, 2006; Lauber et al., 2009). However, the exact mechanisms by which pH influences soil microbial diversity is not well understood.

Two general theories have been suggested. First, that pH directly affects the survival of cells with narrow pH tolerances (Madigan et al., 1997; Fierer and Jackson, 2006). Second, that soil pH indirectly impact microbial diversity by influencing the chemical form, concentration, and availability of key nutrients, which in turn, influences cell activity and survival (Kemmitt et al., 2006). Here we show soil P level displayed a significant linear relationship with both diversity and richness. Therefore, we suggest this is best explained by an indirect mechanism of nutrient-limitation, specifically P, by which soil pH is affecting overall diversity and richness. This is noteworthy because such a relationship between P and diversity was not seen by Chu et al. (2010) in their survey of more acidic Arctic soils.

## Conclusion

In this study, we have shown that bacterial community structure and diversity in the Kongsfjorden polar deserts is similar at the phylum level to that of other Arctic tundra and global soils. The notable exception to previous community compositions reported is the *Acidobacteria* are only a minor component of the bacterial community relative to their representation in other Arctic soils that is consistent with the generally higher soil pH in this landscape. Further, Archaea comprise only a small proportion of the total prokaryotic community in our polar desert soils. We also have shown that soil pH, P, and Ca are drivers of β-diversity in the Kongsfjorden polar deserts and are correlated with overall diversity and richness. These results support previous work that showed bacterial abundances in these soils are constrained by P-supply controlled by lithologically driven pH variation. Given the greatest effects of global warming are expected to have the largest impact in polar latitudes, such observations are critical to understanding controls on Arctic microbial communities, which will allow us to better predict their response to a warming climate.

## Author Contributions

Arctic field work was performed by CM, NG, JR, and DG. Supporting laboratory analysis was done by CM and JR. Preparation of samples for DNA sequencing was done by CM, whereas bioinformatics analysis was performed by MW and CM. Integrated data analysis was done by CM, NG, CH, and DG. The draft manuscript was prepared by CM, MW, and NG, where revisions were performed by all authors. Final preparation for submission was done by CM and DG.

## Conflict of Interest Statement

The authors declare that the research was conducted in the absence of any commercial or financial relationships that could be construed as a potential conflict of interest.
