# Supplementary material for: Microbial Communities in a High Arctic Polar Desert Landscape
Source: Front Microbiol. 2016 Mar 31;7:419. doi: 10.3389/fmicb.2016.00419 (PMC4814466; doi:10.3389/fmicb.2016.00419)
Supplement: Supplementary file 1 [file Data_Sheet_1.PDF]

Microbial communities in a High Arctic polar desert landscape

Supplementary Material

*Clare M. McCann[1], Matthew Wade[1], Neil D. Gray [1], Jennifer A. Roberts [2], Casey R.J. Hubert [1,3] and David W. Graham[1]**

[1] Newcastle University, School of Civil Engineering and Geosciences, Newcastle upon Tyne, United Kingdom NE1 7RU

[2] University of Kansas, Department of Geology, Lawrence, KS, USA 66045

[3]University of Calgary, Energy Bioengineering and Geomicrobiology, Calgary, AB, Canada T2N 1N4

*corresponding author e-mail: david.graham@newcastle.ac.uk; telephone: +44 (0) 191 208 7930

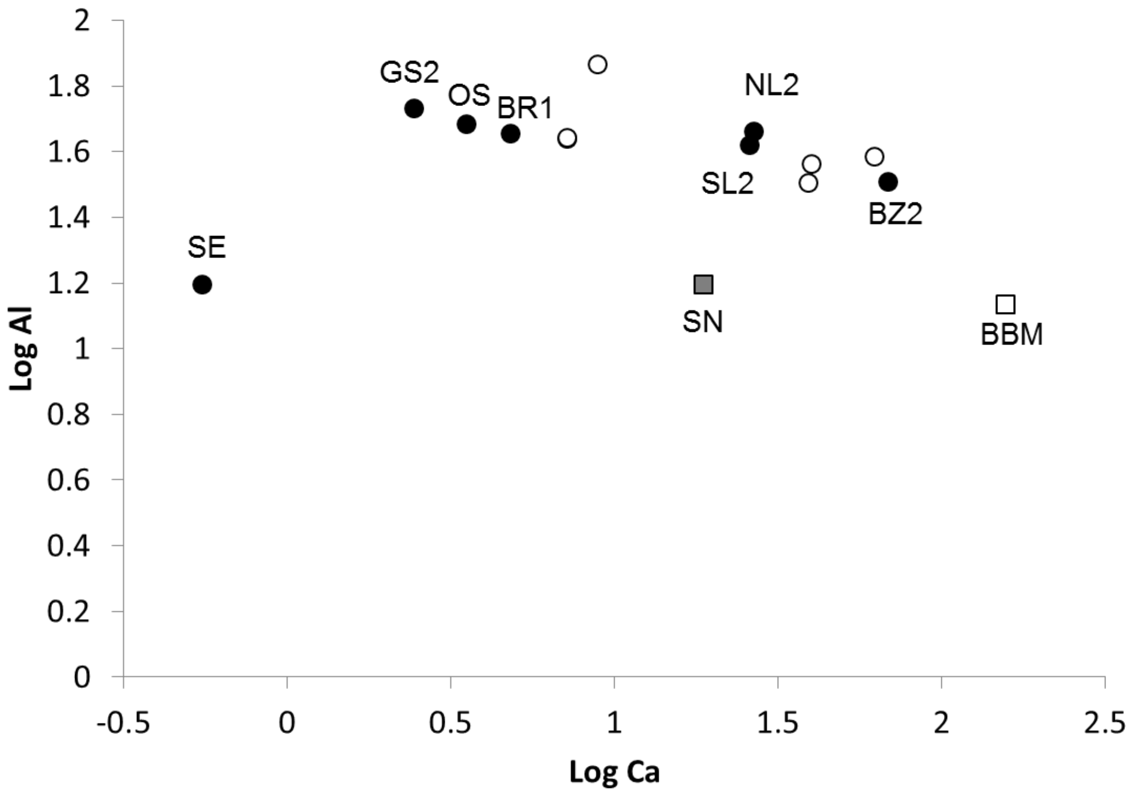

Supplementary Figure 1: Geochemical distributions of the soils selected for this study in relation to the regional soil geochemistry across Kongsfjorden, Svalbard, which are formed from the differential weathering of metamorphic carbonate [represented by Ca] and aluminosilicate rocks [represented by Al] (Gray *et al.*, 2014). Closed circles represent the polar desert soils selected for this study. Squares represent the the glacial moraine, and the grey square the tundra soil not reported in Gray *et al,.* (2014) Open circles represent the other sites reported in Grey *et al.,* (2014) which were not included in this study.

Characteristics of the tundra soil. Solvatnet (SN) displayed a circumneutral pH and had higher levels of moisture, organic matter, C, N and P, in comparison to the other soils of this study (Supplementary Table 1). Increased nutrients are a consequence of inputs from decomposing moss (mainly *Calliergon richardsonii)* and grass (*Dupontia pelligera)*, in addition to droppings from birds (mainly Barnacle Geese, *Branta leucopsis*) and Svalbard reindeer (*Rangifer tarandus plathyrynchus*) (Høj *et al.*, 2005; Tveit *et al.*, 2013; Zwolicki *et al.*, 2013). Unlike the other soils in the region, SN is not N-limited, showing a low molar C:N ratio (2 ± 0). In contrast, the molar C:P ratios were high, (1060 ± 0) indicating significant P limitation, inherent in all of the Kongsfjord soils, with SN the most P limited of all.

XRD analysis of the organic soil confirmed the presence of quartz [SiO_2], calcite $CaCO_3$, dolomite [$CaMg(CO_3)_2$], smectites, kaolinite [$Al_2Si_2O_5(OH)_4$] and mixed valence iron oxyhydroxides ($HFeO_2$). This is consistent with the other Kongsfjorden soils which are composed of primary and secondary carbonates in conjunction with inputs of weathered clay and oxide minerals from mica schists (Mann *et al.*, 1986; Gray *et al.*, 2014). When compared to the eight mineral soils, SN showed lower levels of Mg and Al relative to Ca, reflective of dolomite and clays/iron oxides (Supplementary Figure 2a and 1c), most likely the result of a dilution effect due to the high organic component of the soil. Nonetheless, SN fitted to patterns observed for combinations of clay minerals, and mixed valence iron oxyhydroxides (Supplementary Figure 2d) and the previously reported strong relationship between soil pH and carbonate minerals in the area (Supplementary Figure 2b).

Supplementary Table 1: Characteristics of the tundra soil, Solvatnet (SN)

Analyte	
**Longitude and latitude**	78°55'54.1, 11°56.60.8
**Non-metal characteristics**	
**pH**	6.91 ± 0.06
**Moisture**[a]	697 ± 33.24
**TOC**[b]	37.6 ± 0.63
**TKN**[b]	17.3 ± 0.00*
**NH_4^{+}**[b]	12.2 ± 2.12
**NO_3^{-}**[b]	3.63 ± 0.53
**P**[c]	35.5 ± 0.0*
**$\delta\ ^{13}C_{DIC}$**[d]	-16.4
**Soil Metals**	
**Ca**[e]	18.8 ± 0.82
**Mg**[e]	4.03 ± 0.16
**Al**[e]	15.7 ± 0.04
**K**[e]	6.96 ± 0.01
**Fe**[e]	2.30 ± 0.26
**Cu**[f]	24.5 ± 0.31
**16S rRNA gene abundance**[g]	
**Total Bacteria**	9.51 ± 0.11
**Type I Methanotrophs**	8.06 ± 0.15
**Type II Methanotrophs**	6.91 ± 0.02

Notes: [a] % dry weight soil ± standard error.

[b] mmol $g^{-1 dry\ soil}$ ± standard error.

[c] µmol $g^{-1 dry\ soil}$ ± standard error.

[d] **δ 13C** of soil water dissolved organic carbon in VDPB (‰).

[e] g $kg^{-1 dry\ soil}$ ± standard error. The standard errors were derived from replicated ICP analysis of acid digests of individual bulk soil samples.

[f] mg $kg^{-1 dry\ soil}$ ± standard error. The standard errors were derived from replicated ICP analysis of acid digests of individual bulk soil samples.

[g] log 16S rDNA gene abundances $g^{-1 dry\ soil}$ ± standard error.

* Due to low sample mass only for one replicate to be analysed

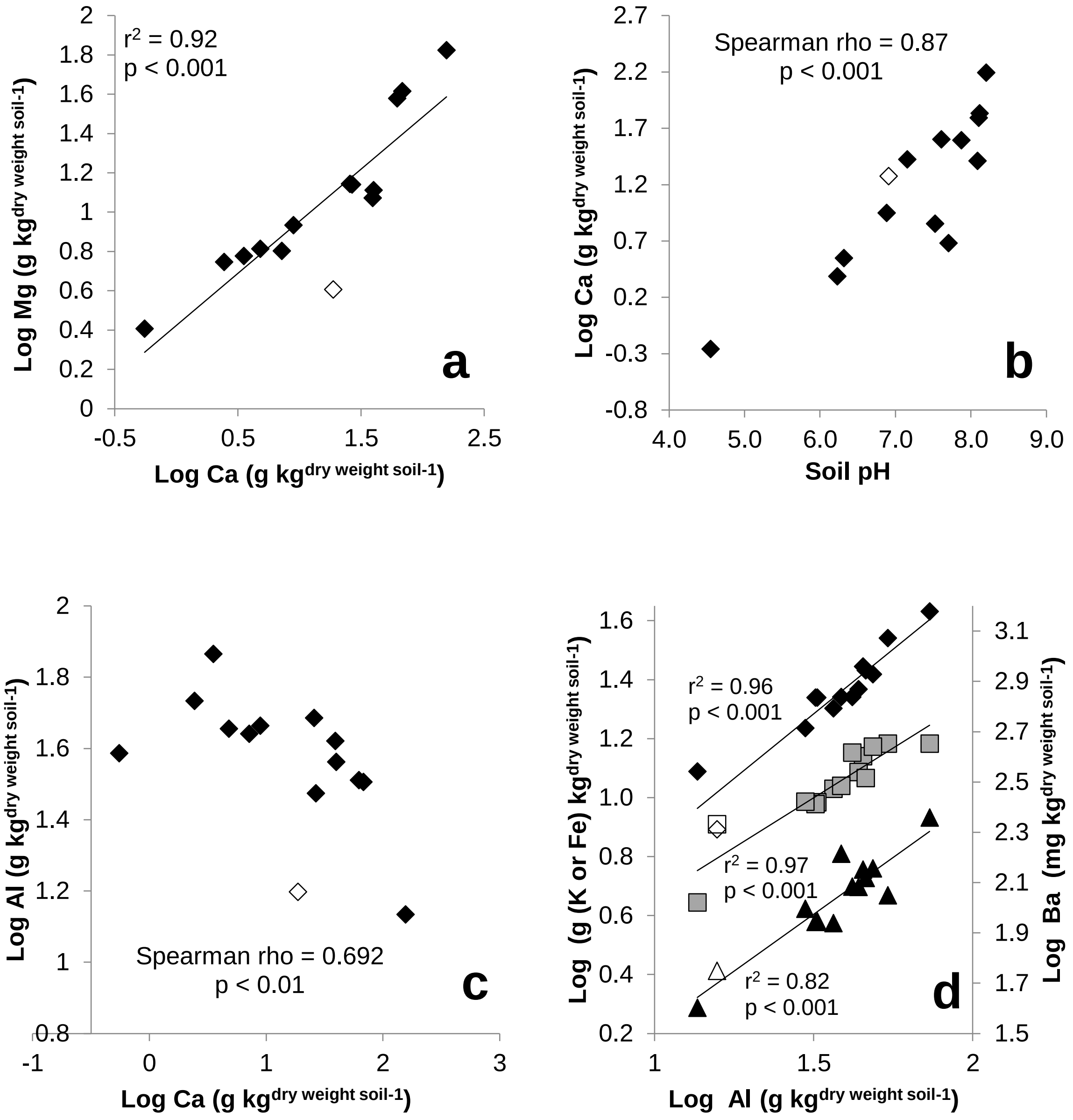

Supplementary Figure 2: Bivariate correlations of metal compositions and pH indicative of the distributions of dolomite and clay minerals across the 13 mineral Kongsfjord soils reproduced from Gray *et al.*(2014) including solvatnet (SN) (open symbol on each graph) (a) Crossplot of Ca and Mg. (b) Crossplot of Ca and pH. (c) Crossplot of Ca and Al. (d) Crossplots of Al with K (diamonds), Fe (triangles) and Ba (squares). The p values indicate the significance of linear (Pearson, r^2) or non-linear (Spearman) correlation coefficients for pairs of variables (SPSS Statistics version 21; IBM).

Supplementary Table 2: Relative abundance of bacterial and archaeal phyla across all the Kongsfjorden soils and for each individual sampling location. Numbers in parenthesis show the respective rank abundances derived from the proportional abundance of sequences for the top 10 phyla.

	All	Polar desert							Tundra	Glacial Moraine
		BZ2	BR1	GS2	NL2	SE	SL2	OS	SN	BBM
Proteobacteria	33.9 (1)	32.0 (1)	27.3 (1)	35.8 (1)	23.9 (2)	46.9 (1)	31.1 (1)	26.7 (2)	29.4 (1)	51.9 (1)
Actinobacteria	20.5 (2)	18.0 (2)	22.7 (2)	18.4 (2)	36.4 (1)	14.1 (2)	11.3 (5)	29.9 (1)	16.9 (2)	16.5 (2)
Chloroflexi	11.8 (3)	14.6 (3)	20.2 (3)	8.87 (3)	11.2 (3)	7.54 (4)	14.3 (3)	13.0 (3)	13.8 (3)	2.88 (5)
Bacteroidetes	6.86 (4)	7.49 (4)	3.92 (6)	6.37 (5)	3.25 (7)	1.72 (10)	12.7 (4)	2.66 (8)	12.6 (4)	11.0 (3)
Acidobacteria	5.17 (5)	5.00 (7)	9.37 (4)	5.60 (6)	6.00 (4)	7.17 (5)	1.01 (10)	6.95 (4)	3.71 (8)	1.72 (7)
Planctomycetes	4.60 (6)	7.31 (5)	2.70 (7)	7.49 (4)	4.50 (6)	7.56 (3)	2.75 (7)	4.23 (6)	3.71 (9)	1.16 (10)
Cyanobacteria	3.70 (7)	6.11 (6)	0.85	0.54	2.28 (8)	2.25 (9)	16.9 (2)	2.57 (9)	0.24	1.54 (8)
Gemmatimonadetes	3.61 (8)	2.65 (8)	4.66 (5)	4.47 (7)	5.53 (5)	2.94 (7)	2.43 (8)	4.62 (5)	3.78 (7)	1.36 (9)
Firmicutes	2.49 (9)	0.56	1.51 (9)	3.21 (9)	0.75	2.45 (8)	0.47	1.13	3.98 (6)	8.37 (4)
Verrucomicrobia	2.33 (10)	2.63 (9)	1.90 (8)	3.49 (8)	1.52 (9)	0.38	1.50 (9)	3.07 (7)	4.54 (5)	1.97 (6)
Other (Bacteria)	1.53	1.38 (10)	1.39 (10)	1.34 (10)	0.85	0.94	2.90 (6)	1.66 (10)	3.03(10)	0.31
Nitrospirae	0.67	0.76	1.27	1.20	1.21 (10)	0.01	0.00	0.94	0.61	0.07
Chlorobi	0.45	0.22	0.30	0.49	0.13	0.08	0.44	0.55	1.56	0.31
WPS-2	0.40	0.00	0.01	0.02	0.01	3.46 (6)	0.05	0.02	0.00	0.00
Armatimonadetes	0.35	0.36	0.28	0.92	0.40	0.19	0.07	0.33	0.22	0.33
Cren- and Thaumarchaeota	0.25	0.25	0.55	0.09	1.18	0.00	0.00	0.11	0.12	0.00
WS3	0.21	0.15	0.21	0.02	0.38	0.00	0.61	0.24	0.24	0.03
TM7	0.19	0.02	0.00	0.27	0.03	0.99	0.05	0.17	0.17	0.00
AD3	0.17	0.00	0.00	0.02	0.00	1.03	0.00	0.44	0.00	0.00

Supplementary Table 2 continued: Relative abundance of bacterial and archaeal phyla across all the Kongsfjorden soils and for each individual sampling location. Numbers in parenthesis show the respective rank abundances derived from the proportional abundance of sequences for the top 10 phyla.

	All	Polar desert							Tundra	Glacial Moraine
		BZ2	BR1	GS2	NL2	SE	SL2	OS	SN	BBM
OP8	0.15	0.01	0.21	0.02	0.00	0.00	1.01	0.00	0.07	0.00
Elusimicrobia	0.12	0.14	0.10	0.21	0.14	0.05	0.05	0.22	0.17	0.00
Euryarchaeota	0.07	0.00	0.01	0.27	0.00	0.00	0.07	0.07	0.22	0.03
WS4	0.05	0.05	0.01	0.27	0.03	0.00	0.00	0.07	0.02	0.03
TM6	0.05	0.09	0.07	0.11	0.04	0.04	0.00	0.02	0.07	0.00
OD1	0.04	0.00	0.04	0.05	0.03	0.04	0.00	0.07	0.15	0.00
BRC1	0.04	0.02	0.05	0.05	0.00	0.00	0.00	0.00	0.05	0.18
WS1	0.03	0.00	0.03	0.01	0.00	0.00	0.07	0.00	0.02	0.13
OP3	0.03	0.00	0.03	0.09	0.01	0.01	0.00	0.09	0.02	0.00
Spirochaetes	0.03	0.01	0.03	0.04	0.00	0.00	0.05	0.02	0.10	0.00
NC10	0.03	0.00	0.08	0.05	0.00	0.00	0.00	0.00	0.10	0.00
Caldiserica	0.02	0.00	0.00	0.00	0.00	0.00	0.00	0.00	0.22	0.00
NKB19	0.02	0.04	0.07	0.01	0.03	0.00	0.00	0.02	0.00	0.00
OP11	0.02	0.00	0.01	0.02	0.03	0.00	0.02	0.04	0.02	0.02
Fibrobacteres	0.02	0.04	0.01	0.05	0.01	0.00	0.00	0.00	0.00	0.05
Chlamydiae	0.01	0.00	0.00	0.01	0.01	0.04	0.00	0.02	0.00	0.02
WYO	0.01	0.01	0.00	0.06	0.01	0.00	0.00	0.00	0.00	0.00
WS2	0.01	0.01	0.02	0.00	0.01	0.00	0.00	0.00	0.02	0.00
Thermi	0.01	0.00	0.00	0.00	0.00	0.00	0.02	0.00	0.00	0.03

Supplementary Table 2 continued: Relative abundance of bacterial and archaeal phyla across all the Kongsfjorden soils and for each individual sampling location. Numbers in parenthesis show the respective rank abundances derived from the proportional abundance of sequences for the top 10 phyla.

	All	Polar desert							Tundra	Glacial Moraine
		BZ2	BR1	GS2	NL2	SE	SL2	OS	SN	BBM
OP9	0.01	0.00	0.00	0.00	0.00	0.05	0.00	0.00	0.00	0.00
**Other (unclassified)**	0.01	0.00	0.00	0.00	0.00	0.00	0.05	0.00	0.00	0.00
AC1	0.01	0.00	0.00	0.00	0.00	0.00	0.05	0.00	0.00	0.00
GN02	0.00	0.00	0.02	0.00	0.00	0.00	0.00	0.00	0.02	0.00
GAL15	0.00	0.01	0.01	0.00	0.01	0.00	0.00	0.00	0.00	0.00
Lentisphaerae	0.00	0.01	0.02	0.00	0.00	0.00	0.00	0.00	0.00	0.00
FCPU426	0.00	0.00	0.00	0.00	0.00	0.00	0.02	0.00	0.00	0.00
WS5	0.00	0.00	0.00	0.01	0.00	0.00	0.00	0.00	0.00	0.00
Fusobacteria	0.00	0.01	0.00	0.00	0.00	0.00	0.00	0.00	0.00	0.00
GOUTA4	0.00	0.01	0.00	0.00	0.00	0.00	0.00	0.00	0.00	0.00

Supplementary Table 3: Relative abundance (%) of the most dominant genra within the *Cyanobacterial* phylum in Kongsfjorden soils.

Class	Order	Family	Genus	All	Polar desert	Tundra	Glacial ***M***oraine
Synechococcophycideae	*Pseudanabaenales*	*Pseudanabaenaceae*	*Leptolyngbya*	30.3	33.9	0.00	3.94
Synechococcophycideae	*Pseudanabaenales*	*Pseudanabaenaceae*	-	20.0	20.0	2.70	18.2
Synechococcophycideae	*Synechococcales*	*Synechococcaceae*	*Prochlorococcus*	16.4	21.6	0.00	0.00
Synechococcophycideae	*Synechococcales*	*Synechococcaceae*	*Other*	12.0	13.2	0.00	0.00
Synechococcophycideae	*Synechococcales*	*Synechococcaceae*	*Arthronema*	11.4	12.8	0.00	0.00
Chloroplast	*Stramenopiles*	-	-	9.11	9.49	15.4	1.05
Oscillatoriophycideae	*Chroococcales*	Other	Other	5.78	6.25	0.00	0.00
Chloroplast	*Stramenopiles*	-	-	5.55	3.44	0.00	31.5
Nostocophycideae	*Nostocales*	*Nostocaceae*	-	4.71	5.06	0.00	0.00
Oscillatoriophycideae	*Chroococcales*	*Gomphosphaeriaceae*	*Snowella*	4.50	4.92	0.00	0.00
Gloeobacterophycideae	*Gloeobacterales*	*Gloeobacteraceae*	*Gloeobacter*	3.53	3.78	0.00	0.00
Oscillatoriophycideae	*Chroococcales*	*Phormidiaceae*	*Phormidium*	3.41	3.18	0.00	8.11
Synechococcophycideae	*Pseudanabaenales*	*Pseudanabaenaceae*	*Pseudanabaena*	2.70	3.14	0.00	1.18
S15B-MN24	-	-	-	1.60	1.30	12.50	3.08
Oscillatoriophycideae	*Chroococcales*	*Cyanobacteriaceae*	-	1.49	1.47	0.00	1.82
Other	-	-	-	1.38	1.38	10.00	0.00
Chloroplast	*Streptophyta*	-	-	1.25	1.24	9.09	0.00
Oscillatoriophycideae	*Chroococcales*	*Xenococcaceae*	Other	0.82	0.37	0.00	8.82
Synechococcophycideae	*Synechococcales*	*Chamaesiphonaceae*	-	0.62	0.14	0.00	2.01
Chloroplast	*Chlorophyta*	-	-	0.41	0.30	7.69	1.08
4C0d-2	*SM1D11*	-	-	0.17	0.02	22.22	-

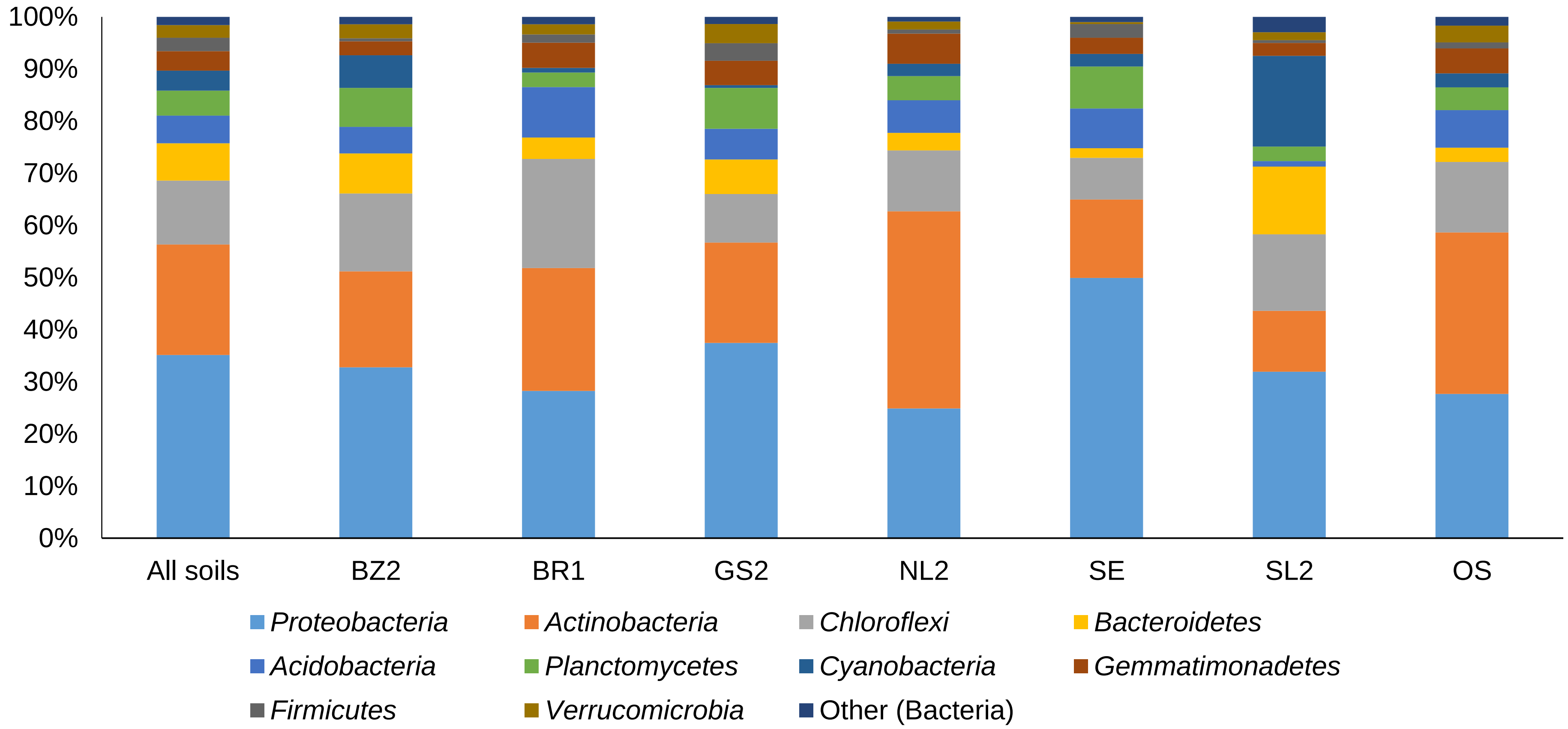

Supplementary Figure 3: Relative abundances of the dominant bacterial phyla in all soils in this study (n = 9), All abundances are based upon the proportional frequency of sequences that could be identified at the phyla level.

Supplementary Table 4: Taxonomic and Phylogenetic Diversity metrics of Kongsfjorden soils[a]

Soil type	Site	Shannon (H')	Chao 1 (S_{chao})	Equitability (E_D)	Observed Species (S)	Faith (PD)
**Polar deserts**	BZ2	9.20 ± 0.01	2520 ± 53.3	0.89 ± 0.00	1260 ± 5.65	99.7 ± 0.27
	OS	8.63 ± 0.00	2210 ± 22.1	0.86 ± 0.00	1060 ± 1.99	87.7 ± 0.16
	NL2	8.52 ± 0.01	2040 ± 34.5	0.85 ± 0.00	1010 ± 4.19	83.7 ± 0.35
	BR1	8.37 ± 0.01	1890 ± 53.1	0.85 ± 0.00	947 ± 7.30	80.0 ± 0.53
	SL2	8.29 ± 0.00	2180 ± 16.4	0.83 ± 0.00	1005 ± 1.90	92.0 ± 0.16
	GS2	8.27 ± 0.01	1650 ± 10.8	0.84 ± 0.00	925 ± 2.65	80.6 ± 0.41
	SE	6.66 ± 0.01	751 ± 16.4	0.76 ± 0.00	423 ± 2.67	42.2 ± 0.30
**Peatland**	SN	8.70 ± 0.00	2150 ± 12.7	0.86 ± 0.00	1070 ± 1.73	92.4 ± 0.18
**Glacial moraine**	BBM	6.52 ± 0.01	731 ± 11.7	0.74 ± 0.00	459 ± 2.64	46.5 ± 0.28

[a] Mean and 1 x standard error of all metrics was based upon 10 random iterations of the sequencing dataset at a sequence per sample depth of 3,946.

Supplementary Table 5: Summary of richness indicators and sampling effort required to capture 90 % of the true diversity in high Arctic soils.

Soil type	Sample	OTUs[a]	Chao[b]	Bayesian parametric estimate[c]	454 Sequencing runs to retrieve 90% of OTUs[d]
**Polar desert**	BR1	2600	1888	4841	10
	BZ2	3088	2522	7912	28
	GS2	1727	1654	3374	10
	SE	710	751	2223	49
	NL2	1814	2036	4306	14
	OS	1468	2208	5376	33
	SL2	1198	2180	4998	35
**Peatland**	SN	1360	2146	4765	30
**Glacial moraine***	BBM	711	731	2595	464

[a] The number of OTUs observed at the 97 % sequence similarity
[b] The Chao non-parametric estimate of total diversity
[c] The median distribution of richness from the best fitting taxa abundance distributions (see Supplementary Table 4)
[d] The corresponding sampling effort i.e. no. of additional 454 runs, based upon the current sample size, required to observe 90 % of the true richness (see Supplementary Table 4 and 5)
*total diversity estimates for BBM indicate that diversity has not been adequately captured and is significantly underestimated in the glacial moraine, with 464 additional sequencing runs required for determination

Supplementary Table 6: Diversity estimates from fits of log-normal, inverse Gaussian and Sichel abundance distributions for high arctic soils. Best fits are highlighted in bold.

Sample	Log-normal	Inverse Gaussian	Sichel
BBM	**1748[a] : 2595[b] : 5061[c]**	1241 : 1589 : 2339	1052 : 1252 : 1594
BR1	6794 : 8461 : 9993	5407 : 6251 : 7137	**4309 : 4841 : 5601**
BZ2	7821 : 9398 : 10878	**7113 : 7912 : 9007**	18431 : 20581 : 22392
GS2	4646 : 5793 : 7654	4028 : 4744 : 5708	**2947 : 3374 : 4052**
SE	2721 : 4480 : 10450	**1550 : 2223 : 4232**	1377 : 1975 : 3337
NL2	6200 : 8329 : 11526	5399 : 7278 : 8486	**3609 : 4306 : 5447**
SN	5378 : 7613 : 10790	4663 : 5989 : 7455	**3539 : 4765 : 7043**
OS	6850 : 9309 : 12771	5423 : 6885 : 8833	**4095 : 5376 : 7561**
SL2	8019 : 14648 : 20982	5514 : 6780 : 7905	**3745 : 4998 : 6609**

[a] Lower 95 % confidence interval
[b] Median diversity
[c] Upper 95 % confidence interval

Supplementary Table 7: Sampling effort estimation for obtaining 90% of the taxonomic diversity based on the taxa abundance distributions (TADs).

Sample	Log-normal	Inverse Gaussian	Sichel
BBM	$5.51 \times 10^5 : 2.81 \times 10^6 : 4.76 \times 10^7$	$6.80 \times 10^4 : 1.28 \times 10^5 : 3.24 \times 10^5$	$3.46 \times 10^4 : 5.55 \times 10^4 : 1.05 \times 10^5$
BR1	$1.48 \times 10^6 : 3.45 \times 10^6 : 6.86 \times 10^6$	$2.84 \times 10^5 : 4.07 \times 10^5 : 5.64 \times 10^5$	$1.23 \times 10^5 : 1.68 \times 10^5 : 2.54 \times 10^5$
BZ2	$7.91 \times 10^5 : 1.54 \times 10^6 : 2.71 \times 10^6$	$2.72 \times 10^5 : 3.55 \times 10^5 : 4.85 \times 10^5$	$2.80 \times 10^6 : 4.12 \times 10^6 : 6.03 \times 10^6$
GS2	$5.61 \times 10^5 : 1.29 \times 10^6 : 3.72 \times 10^6$	$1.69 \times 10^5 : 2.54 \times 10^5 : 3.97 \times 10^5$	$5.84 \times 10^4 : 8.24 \times 10^4 : 1.33 \times 10^5$
SE	$4.88 \times 10^6 : 4.33 \times 10^7 : 2.00 \times 10^9$	$1.53 \times 10^5 : 3.60 \times 10^5 : 1.51 \times 10^6$	$9.62 \times 10^4 : 2.47 \times 10^5 : 9.88 \times 10^5$
NL2	$8.48 \times 10^5 : 2.29 \times 10^6 : 7.33 \times 10^6$	$2.55 \times 10^5 : 4.93 \times 10^5 : 7.12 \times 10^5$	$6.55 \times 10^4 : 9.93 \times 10^4 : 1.73 \times 10^5$
SN	$5.23 \times 10^5 : 1.63 \times 10^6 : 5.35 \times 10^6$	$1.69 \times 10^5 : 3.01 \times 10^5 : 4.91 \times 10^5$	$6.21 \times 10^4 : 1.21 \times 10^5 : 3.09 \times 10^5$
OS	$1.04 \times 10^6 : 2.92 \times 10^6 : 8.95 \times 10^6$	$2.36 \times 10^5 : 4.03 \times 10^5 : 6.89 \times 10^5$	$8.18 \times 10^4 : 1.51 \times 10^5 : 3.24 \times 10^5$
SL2	$2.41 \times 10^6 : 1.69 \times 10^7 : 6.00 \times 10^7$	$3.28 \times 10^5 : 5.19 \times 10^5 : 7.45 \times 10^5$	$7.95 \times 10^4 : 1.43 \times 10^5 : 2.58 \times 10^5$

Supplementary Table 8: Deviance Information Criterion for each taxa abundance distribution (TAD). Lowest values indicate best fit.

Sample	Log-normal	Inverse Gaussian	Sichel	Adding 1 to Sichel
BBM	164	165	163	164
BR1	248	255	247	248
BZ2	193	192	197	198
GS2	179	185	178	179
SE	199	195	196	197
NL2	156	167	154	155
SN	109	112	107	108
OS	122	125	117	118
SL2	124	142	122	123

References:

Gray, N.D., McCann, C.M., Christgen, B., Ahammad, S.Z., Roberts, J.A. and Graham, D.W. (2014) 'Soil geochemistry confines microbial abundances across an arctic landscape; implications for net carbon exchange with the atmosphere', *Biogeochemistry*, 120(1-3), pp. 307-317.

Høj, L., Olsen, R.A. and Torsvik, V.L. (2005) 'Archaeal communities in High Arctic wetlands at Spitsbergen, Norway (78°N) as characterized by 16S rRNA gene fingerprinting', *FEMS Microbiology Ecology*, 53(1), pp. 89-101.

Mann, D.H., Sletten, R.S. and Ugolini, F.C. (1986) 'Soil development at Kongsfjorden, Spitsbergen', *Polar Research*, 4(1), pp. 1-16.

Tveit, A., Schwacke, R., Svenning, M.M. and Urich, T. (2013) 'Organic carbon transformations in high-Arctic peat soils: key functions and microorganisms', *ISME J*, 7(2), pp. 299-311.

Zwolicki, A., Zmudczyńska-Skarbek, K.M., Iliszko, L. and Stempniewicz, L. (2013) 'Guano deposition and nutrient enrichment in the vicinity of planktivorous and piscivorous seabird colonies in Spitsbergen', *Polar Biology*, 36(3), pp. 363-372.
